# Acute exposure of Nicotine during *Drosophila* puncture injury activates an epidermal wound response reaction.

**DOI:** 10.17912/micropub.biology.000415

**Published:** 2021-07-02

**Authors:** Chrissy Cherenfant, Michelle T Juarez

**Affiliations:** 1 City University of New York School of Medicine; 2 City College of New York; 3 Howard Hughes Medical Institute, Science Education

## Abstract

Epidermal wound reaction after injury can be visualized in *Drosophila melanogaster* embryos by transgenic fluorescent wound reporters. A “local” reaction is limited to the epidermal cells surrounding a wound site occurs in *wildtype* embryos. A “global” reaction extends beyond the wound site to all epidermal cells occurs in immune response mutants (e.g. *Flotillin-2 *and* Toll*). The aim of this investigation is to explore the effect of nicotine on the localization of *Drosophila *wound reaction. Nicotine may have the potential to negatively affect wound repair by inhibiting a local reaction, and ultimately impeding epidermal wound recovery in *Drosophila*. We find nicotine exposure activates a global reaction after puncture injury of *Drosophila *embryos*.* Determining the effect of nicotine exposure on wound reaction in *Drosophila *may lead to improved understanding of how nicotine use in humans may influence wound healing after tissue damage.

**Figure 1. Acute exposure of nicotine effects on Ddc-GFP epidermal wound response reporter activity:**
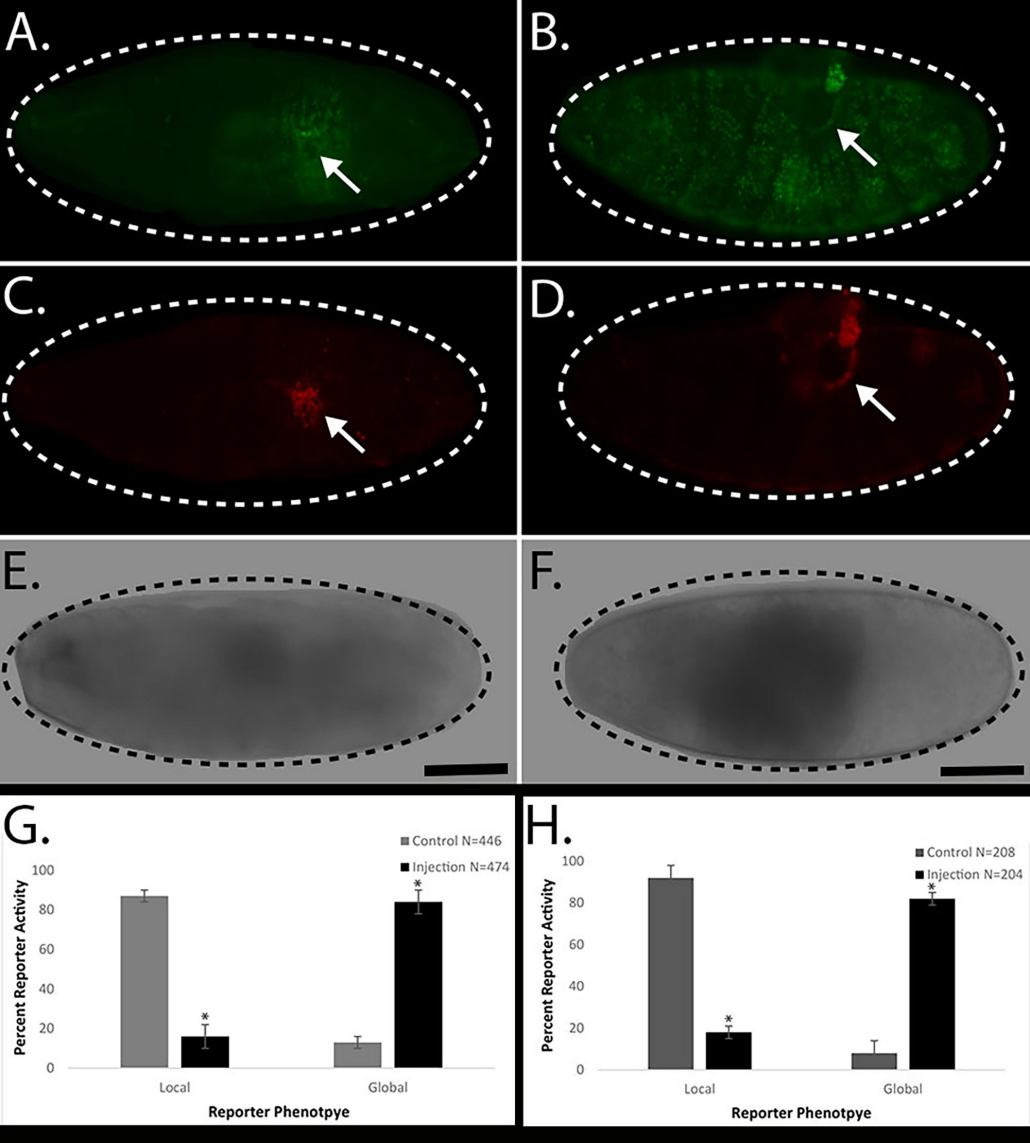
Fluorescence images of control embryos (A, C) and nicotine solution injection embryos (B, D). *Ddc*-GFP phenotype in control embryos is “local” and restricted to the site of injury (A). *Ddc*-GFP phenotype in nicotine solution injection embryos is “global” and spread throughout all epidermal cells (B). Ethidium homodimer III stains necrotic tissue and is restricted to the site of injury in both control and nicotine solution injection embryos (C, D). Embryonic development of a standard collection method shows normal patterning at stage 16 (E). Embryonic development of a nicotine exposure collection method shows termination of development despite being the same age as the standard collection method (F). Dashed lines mark the outlines of the embryo. Arrows mark the site of injury. Solid black lines mark the scale bar of 50µm. Quantification of percent reporter activity (G, H). Averages of the *Ddc*-GFP reporter activity were determined by number of each phenotype divided by the total number for each nicotine solution experiment (G). The standard deviation for the combined puncture experiments is +/- 3. The standard deviation for the combined nicotine injection experiments is +/- 6. The two tailed P value is less than 0.0001. Averages of the *Ddc*-GFP reporter activity were determined by number of each phenotype divided by the total number for each e-cigarette solution experiment (H). The standard deviation for the combined puncture experiments is +/- 6. The standard deviation for the combined e-cigarette injection experiments is +/- 4. The two tailed P value is less than 0.0001.

## Description

Biological model organisms provide an excellent *in vivo* system to test the effects of chemical exposure on developmental pathways. Recent studies in fruit fly *Drosophila melanogaster* highlight the strength of combining genetic screens and chemical inhibitors to dissect complex processes that promote an epidermal wound response during embryonic puncture injury assays (Juarez *et al.* 2011). The epidermal wound response reporter is based on the transcriptional reactivation of barrier formation genes, *Dopa Decarboxylase (Ddc)* and *tyrosine hydroxylase (ple*) in a localized pattern of cells that surround the site of injury (Juarez 2016). These genes each display their respective transcriptional expression phenotypes in epidermal cells: local gene activation is limited to the cells surrounding the site of damage in wild type genetic backgrounds and global gene activation is unlimited and spreads beyond the cells surrounding the site of damage in immune response mutants (e.g. *Flotillin-2* and *Toll*) (Juarez *et al.* 2011; Patterson *et al.* 2013). The epidermal wound response reporter is based on the characterization of a transgenic enhancer sequence that activates green fluorescence protein (GFP) in a wound-dependent and epidermal-specific pattern localized in the cells that surround the site of injury, referred to as *Ddc*-GFP (Juarez 2016). Fundamentally, the fluorescent reporter provides an *in-vivo* tool for visualizing the wound reaction caused by epidermal injury.

The goal of this current study is to test the effect of acute nicotine exposure on the activation and localization of an embryonic epidermal wound response reporter in *Drosophila*. The impact of nicotine on models of wound regulation, reaction, and repair can promote our understanding of the barriers to tissue repair and the pathways that promote wound healing (Martin *et al.* 2009). A recent report describes a potential intersection among nicotine and Flottilin-2 mediated lipid rafts during endothelial cell inflammatory response (Peña *et al.* 2011). Using *Drosophila* as a model organism to study drug abuse benefits from the strength of advanced genetic tools, well-conserved molecular pathways, simple behavioral assays, and easily visualized anatomy (Wolf and Heberlein 2003). One advantage of *Drosophila* as an *in vivo* model for nicotine research is the large numbers of samples that can be analyzed to test the specific effects of acute exposure via injection assays or chronic exposure via ingestion assays (Matta *et al.* 2007). Furthermore, *Drosophila*’s use in research continues to provide beneficial insight into mammalian processes. This is due to its euchromatin sequence having high degree of molecular similarity with mammals, where about 75% of human disease-causing genes are believed to have a functional homolog in *Drosophila* (Pandey and Nichols 2011).

Control puncture injury generally produces a local *Ddc*-GFP embryonic wound response reporter phenotype that is restricted to the cells surrounding the site of injury, a “local” wound reaction (Figure 1A). Acute exposure of nicotine in *Drosophila* embryos, via delivery by microinjection, effectively increases the *Ddc*-GFP embryonic wound response reporter phenotype throughout all the epidermal cells, a “global” wound reaction (Figure 1B). To confirm that the acute exposure of nicotine solution was not activating cell death, we stained the control and experimental embryos with a necrosis stain, EthD-III. Previous studies have demonstrated that genetic mutant backgrounds that exhibit “global” activation of *Ddc*-GFP do not inflict widespread necrosis (Patterson *et al.* 2013). Both control puncture injury and experimental microinjection of nicotine solution embryos have localized cell death that is restricted to the cells surrounding the site of injury (Figure 1 C, D). An average summary of 4 experiments showed that control puncture injury embryos demonstrated 87% (388/446) local wound reporter activity and experimental microinjection of nicotine solution embryos produced 85% (401/474) global wound reporter activity (Figure 1G). For comparison, we also tested the effect of acute exposure of E-cigarette solution in *Drosophila* embryos, via delivery by microinjection and we observed similar increases in *Ddc*-GFP embryonic wound reporter phenotype. An average of 4 experiments shows that control puncture embryos produced 93% (194/208) local wound reporter activity and microinjection of E-cigarette solution embryos developed 82% (168/204) global wound reporter activity (Figure 1H). Our results suggest that puncture injury in the presence of acute exposure to nicotine correlates with a global *Ddc*-GFP wound reporter reaction in *Drosophila* embryos.

The use of *Drosophila* as a model system in developing future studies to describe an intersection between the genetic pathways regulating epidermal wound response and nicotine exposure may have the ability to uncover novel functions of well-conserved genes, similar to the recent advances in understanding the role of innate immune responses in coordinating a trypsin-mediated epidermal wound response (Patterson *et al.* 2013; Capilla *et al.* 2017). Another report of *Drosophila* acute and chronic nicotine exposure models highlights the novel role of *escargot* (*esg*), a gene that encodes transcription factors required for central nervous system development, in promoting the behavior and sensitivity response to nicotine (Sanchez-Díaz *et al.* 2015). Interestingly, the developmental stage of *Drosophila* modulates the effects of nicotine exposure. Acute injections of nicotine into larval developmental stages can increase the heart rate however, injections of nicotine into adult flies act to decrease the heart rate (Zornik *et al.* 1999). Not many reports have discussed the specific effects E-cigarette exposure has on animal models. A recent study found the presence of free radicals present in the E-cigarette vapor and showed an impaired inflammatory response in a murine model of wound repair following vaporized E-cigarette exposure (Sussan *et al.* 2015). The ultimate goal of using animal models of tissue repair is not only to understand the coordination of the response to injury, which mediates developmental processes like cell proliferation and tissue remodeling, but to also determine how the regulatory processes respond to diseases (e.g. cancer). For example studies describing the overlap between chronic injury and cancer pathogenesis, provide evidence for the transcriptional regulation as an initiating point for both tissue repair and cancer progression (Schäfer and Werner 2008). In the future, work in model organisms can enhance our understanding of complex biological processes and potentially improve the fight against many health disparities that impact human health (Jones *et al.* 2006). With further pursuit of this investigation, we may gain improved understanding of how external factors and human actions, like smoking, may ultimately affect healing after injury. Using studies in *Drosophila*, as a model organism, will provide new directions for clinical studies to improve recovery in humans following tissue damage.

## Methods

Simultaneous puncture injury and microinjection assays were previously described (Juarez *et al.* 2013). In summary, *Drosophila* embryos are collected and aged to stage 16, aligned on a glass slide and punctured using a microinjection apparatus combined with an inverted microscope. Compound fluorescence microscopy (Leica Biosystems DM5500B) was used to determine the wound reaction as the range of *Dopa Decarboxylase* (*Ddc*) epidermal wound reporter activity by visualizing the restricted fluorescence, “local” *Ddc*-GFP phenotype or the expanded fluorescence, “global” *Ddc*-GFP phenotype. The standard deviation was determined by calculating the square root of the variance between averages observed in the 4 experiments. For example, the nicotine solution microinjection data was collected in 4 independent experiments (exp). The averages of the “local” Ddc-GFP phenotype of the 4 control experiments were: exp1-89% (N=127/143), exp2-89% (N=95/107), exp3-83% (N=69/83), exp4-82% (N=97/113). The variance between the 4 exp was determined by calculating the mean of the squared difference – equal to 10.75. The standard deviation was determined by calculating the square root of the variance – equal to 3. The unpaired t-test was calculated to compare the means of the two groups (1-control and 2-injected). The two-tailed P value is less than 0.0001 for both nicotine exposure as well as E-cigarette exposure. The Graph Pad Software, (La Jolla California USA, www.graphpad.com) was used to determine all statistical values.

## Reagents

The epidermal wound response reporter is based on the characterization of a transgenic *Dopa decarboxylase* (*Ddc*) enhancer sequence that activates green fluorescence protein (GFP) in a wound-dependent and epidermal-specific pattern localized in the cells that surround the site of injury, referred to as *Ddc*-GFP (Juarez 2016). Control microinjection solution was prepared by mixing a 1 to 1 ratio of Toludine Blue 0.5% solution (Fisher Science Catalog #S25613) and water. Nicotine microinjection solution was prepared by mixing a 1 to 1 ratio of Toluidine Blue 0.5% solution (Fisher Science Catalog #S25613) and Nicotine Solution (Fisher Science Catalog #S25446). E-cigarette microinjection solution was prepared by mixing a 1 to 1 ratio of Toluidine Blue 0.5% solution (Fisher Science Catalog #S25613) and E-cigarette solution, 2.4% nicotine (Loillard Technologies, blu-black cartomizers). Ethidium Homodimer III (EthD-III) stain (Biotium, Catalog #30018) was added to both control puncture and experimental microinjection embryos following a two-hour recovery period and 5min permeabilization treatment (1 to 1 heptane and 1X Phosphate Buffered Solution).
